# Identity Threat as a Barrier to NHS Psychology Professionals’ Adoption of Generative AI: A Technology Acceptance Model Study

**DOI:** 10.3390/bs16101827

**Published:** 2026-10-06

**Authors:** Sam Pye, Cassie M. Hazell, Kimberley J. Smith, Aislinn Bergin

**Affiliations:** 1 School of Psychology, University of Surrey, Guildford GU2 7XH, UK; cassie.hazell@surrey.ac.uk (C.M.H.);; 2 NIHR MindTech HealthTech Research Centre, School of Computer Science, University of Nottingham, Nottingham NG7 2RD, UK

**Keywords:** generative artificial intelligence, Technology Acceptance Model, professional identity threat, self-threat, NHS psychology professionals, clinician attitudes, structural equation modelling

## Abstract

Introduction. The development and adoption of generative artificial intelligence (GenAI) technologies is rapidly expanding, but its integration into psychological services depends on clinician acceptance. Little empirical work has examined how identity-related threat shapes psychology professionals’ adoption intentions of GenAI. The present research tested an extended technology acceptance model (TAM), with the addition of personal and professional identity threats, to explain psychology professionals’ adoption intentions of GenAI technologies for clinically relevant usage. Methods. A total of 201 UK psychology professionals completed a cross-sectional online survey. Three sequential structural equation models were tested, incorporating perceived usefulness, perceived ease of use, and social influence as core predictors, with threats to professional capabilities and self-threat added as extensions. Two open-ended qualitative questions were analysed using content analysis to provide broader contextual insight into current use and barriers to adoption. Results. All models demonstrated acceptable fit (CFI = 0.936–0.938; RMSEA = 0.071–0.072). Perceived usefulness emerged as the strongest correlate of adoption intention across all models (β = 0.67–0.69). Threats to professional capabilities and self-threat were each reliably negatively associated with adoption intention when entered separately, above and beyond core TAM predictors (β = −0.20 and β = −0.16 respectively). Social influence exerted a reliable indirect effect on behavioural intention through perceived usefulness across all models, as did perceived ease of use in the two models incorporating identity threat. Content analysis identified ethical concerns, lack of trust, and professional identity threats as prominent barriers to adoption. Discussion. Findings highlight the importance of considering both classic TAM factors and identity-related concerns when developing GenAI and implementing such technology into psychological services.

## 1. Introduction

Generative AI (GenAI) refers to a class of AI technologies that can generate new content (including text, image and audio) by learning patterns from large datasets and responding to user prompts (Lim et al., 2023; He et al., 2025). Prominent publicly available examples of these GenAI tools, often referred to as large language models (LLMs), include ChatGPT (by OpenAI), Gemini (by Google), and Claude (by Anthropic). The sophistication and conversational fluency of these models have resulted in GenAI’s widespread adoption across multiple sectors, including healthcare.

Generative AI technologies are already showing emerging potential within healthcare and psychological services, ranging from administration support, where AI has been used to create discharge summaries and clinic letters (Patel & Lam, 2023; Ali et al., 2023; Schwieger et al., 2024), to applications in clinical decision support (Perlis et al., 2024). Generative AI is also being explored in relation to patient-facing interventions, including chatbot-assisted therapy (Heinz et al., 2025) and therapy-adjunctive tools that deliver personalised therapeutic content between sessions (Habicht et al., 2025). However, alongside these potentially beneficial uses, there could also be significant professional and ethical challenges. For instance, concerns have been raised regarding the accuracy and reliability of AI systems, their transparency and explainability, data privacy, and the potential for algorithmic bias to perpetuate stigma and inequality (Wang et al., 2025; Salah et al., 2024; Blease & Rodman, 2025; Lawrence et al., 2024).

In addition to addressing technological feasibility and ethical issues, the successful integration of GenAI technologies into healthcare hinges on the willingness of professionals to adopt and engage with them (Greenhalgh et al., 2017). Historically, clinician acceptance has been identified as a barrier to the adoption of safe and effective technologies (Cowan et al., 2019). This finding can be understood within the Technology Acceptance Model (TAM, Davis, 1989). TAM provides a well-established framework for predicting users’ intention to adopt new technologies. Adoption behaviour is proposed to be primarily driven by two psychosocial factors: perceived usefulness (i.e., the degree to which a person believes a technology will enhance their performance) and perceived ease of use (i.e., the degree to which they believe using the technology will require little effort).

Since the development of the core model, revised versions of the model have added additional predictors, namely social influence and facilitating conditions, as well as adding indirect pathways demonstrating the indirect effect that perceived ease of use and social influence have on adoption intentions via perceived usefulness (Venkatesh & Davis, 2000; Venkatesh & Bala, 2008). Meta-analyses have demonstrated TAM’s wide applicability across different fields (King & He, 2006). However, there is comparatively little research applying these frameworks to psychological services. Kleine et al. (2023) examined psychology students’ and psychotherapy trainees’ intentions to adopt two AI clinical support tools finding that perceived usefulness and social influence were the strongest predictors of adoption intentions (Kleine et al., 2023). More recently Wagner and Schwind (2025) applied a technology acceptance framework to German-based psychotherapists attitudes to adopt AI, finding that perceived usefulness was the strongest predictor of AI acceptance.

In the context of GenAI adoption, psychology professionals may represent a distinct population. Psychological practice is defined in part by competencies such as empathic communication, reflective practice and psychological formulation (Health and Care Professions Council, 2023). These capabilities, which are central to the professional identity of psychology professionals, have historically been viewed as uniquely human and are widely considered resistant to technological replacement. However, recent research has indicated that GenAI systems can increasingly emulate aspects of capabilities traditionally associated with psychological practice, including emotional awareness, empathic communication (Elyoseph et al., 2023; Scholich et al., 2025) and psychological formulation (L.-H. Hsieh et al., 2024). It is perhaps unsurprising, therefore, that 38% of psychologists surveyed by the American Psychological Association (2025) reported concerns that AI may render some or all of their job duties obsolete.

When a technology begins to replicate the capabilities through which a profession defines its value, adoption may be shaped not only by judgements about that technology’s usefulness or ease of use, but by more fundamental concerns about professional distinctiveness and worth. This tension has generated growing scholarly and public discourse about the potential threat GenAI poses to the roles of psychology professionals (Blease et al., 2024; van Zyl, 2026; Zhang & Wang, 2024). A recent narrative review explored the potential negative consequences of AI encroaching on traditional psychological competencies, including fears of replacement, diminished clinical skill, reduced autonomy, and fragmentation of professional roles (van Zyl, 2026). To date, however, this discourse has remained largely conceptual and the role of professional identity in shaping psychology professionals’ adoption of GenAI has not been examined empirically.

Professional identity refers to an individual’s self-perception within their professional role (Ackerhans et al., 2024; Chreim et al., 2007) and, for healthcare professionals, can encompass facets including autonomy, expertise, and influence (Jussupow et al., 2022; Ackerhans et al., 2024). It plays a crucial role in shaping how professionals respond to new technologies, with prior research showing that clinicians may resist innovations perceived to undermine their autonomy or identity (Walter & Lopez, 2008; Ackerhans et al., 2024; Jussupow et al., 2022). Jussupow et al. (2022) conceptualised threats to professional capabilities as a specific, measurable component of professional identity threat, capturing perceived challenges to professionals’ autonomy, influence, and ability to provide effective care; this construct predicted resistance to AI among medical professionals and students. The same study found that threat to one’s sense of self (self-threat) independently predicted resistance, underscoring the importance of considering personal identity alongside professional identity. This distinction may be especially pertinent for psychology professionals, for whom professional identity is understood to be closely intertwined with the personal self, given the value placed on empathy, authenticity, and self-reflexivity within the role (Schubert et al., 2023; Alves & Gazzola, 2011). Whereas identity threat has recently been modelled as a predictor of AI adoption in other professional groups (Shonhe & Min, 2025), it has not been examined among psychology professionals.

To date, few studies have examined the acceptance of AI tools among psychology professionals in a clinical context (Kleine et al., 2023; Wagner & Schwind, 2025), and no study has examined the role of professional identity threats in shaping adoption intentions. The present study aims to address this gap by applying the TAM to the context of GenAI adoption among UK National Health Service (NHS) psychology professionals, whilst additionally including two forms of identity-related threat: threats to professional capabilities and self-threat. Given the lack of research in this area, the present study additionally includes two open-ended qualitative questions to provide broader contextual insight into psychology professionals’ current uses of and barriers to GenAI adoption in clinical practice.

## 2. Research Model and Hypotheses

The present study tested a series of three sequential extended TAM models using structural equation modelling (SEM) to examine the factors predicting psychology professionals’ behavioural intention to adopt and use GenAI in clinical practice within 3 separate models. In model 1, in line with TAM (Davis, 1989) and TAM2 (Venkatesh & Davis, 2000), we predict that perceived usefulness, perceived ease of use and social influence will positively predict participants’ behavioural intention to adopt GenAI technologies. Drawing on TAM2 (Venkatesh & Davis, 2000), we additionally predict that perceived ease of use and social influence will also predict behavioural intention indirectly through perceived usefulness:

- H1. Perceived Usefulness will positively predict Behavioural Intention to adopt GenAI.
- H2. Perceived Ease of Use will positively predict Behavioural Intention to adopt GenAI directly, and indirectly through Perceived Usefulness.
- H3. Social Influence will positively predict Behavioural Intention to adopt GenAI directly, and indirectly through Perceived Usefulness.

Frequency of clinical use was included as an additional outcome variable to explore whether the predictors of behavioural intention also extended to self-reported behaviour, consistent with the theoretical assumption within TAM that intention predicts usage (Davis, 1989). Consistent with previous research (Jussupow et al., 2022; Walter & Lopez, 2008), Model 2 incorporated threats to professional capabilities, while Model 3 incorporated self-threat as additional predictors. These constructs were entered into separate models to assess their unique contribution.

- H4. Threats to Professional Capabilities will negatively predict Behavioural Intention to adopt GenAI, above and beyond the core TAM predictors.
- H5. Self-Threat will negatively predict Behavioural Intention to adopt GenAI, above and beyond the core TAM predictors.

**Figure 1:**
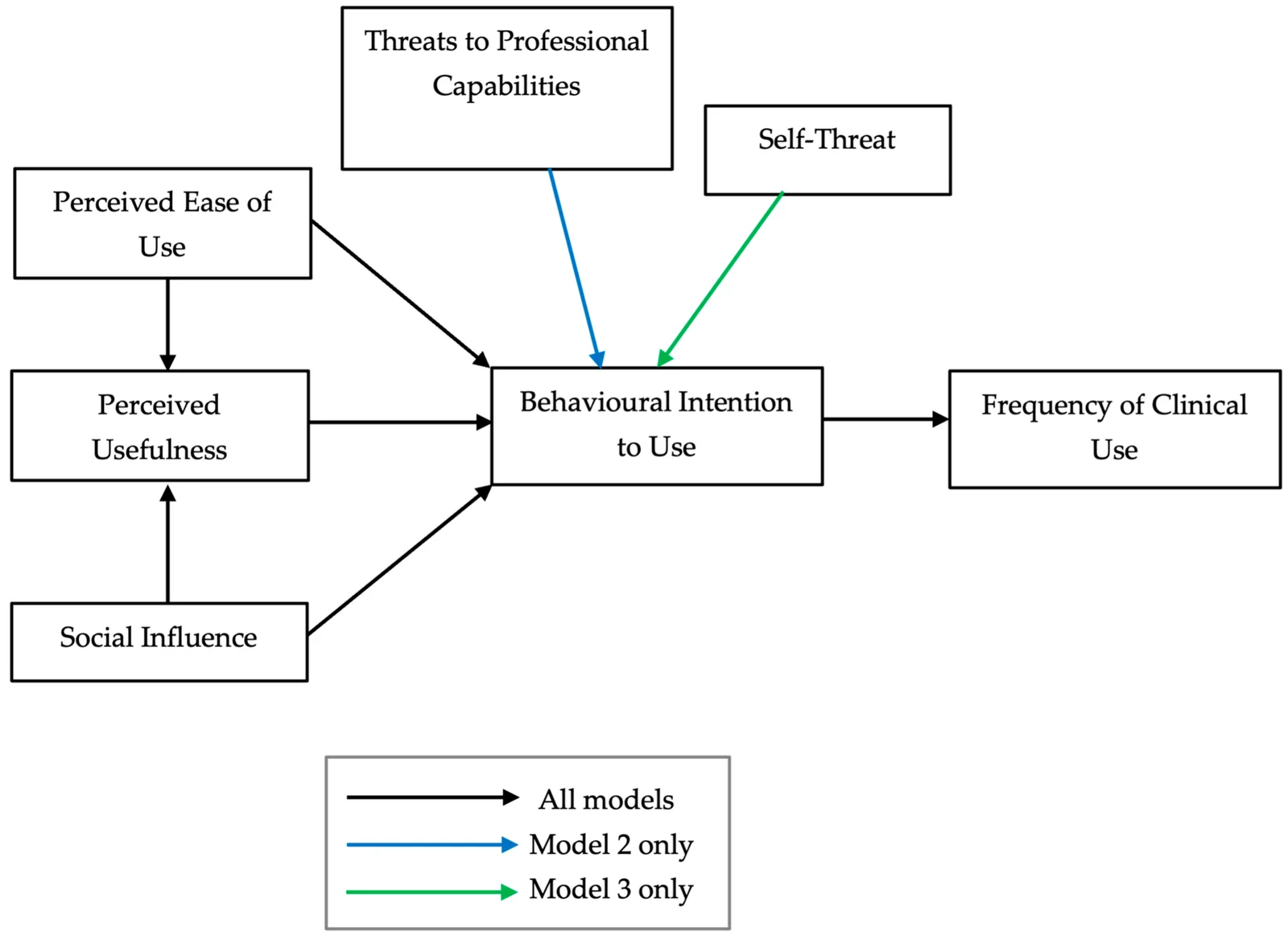
Hypothesis model structure.

The hypothesised model structure is outlined in Figure 1.

## 3. Methods

### 3.1. Study Design

The study employed a mixed-methods cross-sectional design to examine UK NHS psychology professionals’ (including trainees) attitudes toward the adoption of GenAI technologies within clinical practice. Data were collected using an online survey hosted on Qualtrics (Qualtrics, Provo, UT, USA) between March 2025 and July 2025.

### 3.2. Procedure

Before completing the questionnaire, participants were required to read the participant information sheet and provide consent for their participation. The questionnaire began with demographic and profession questions, followed by questions about their familiarity with GenAI and their current usage. To support participants who were unfamiliar with GenAI, a brief description was provided with a definition (taken from Lim et al., 2023; He et al., 2025), giving ChatGPT as an example of a GenAI tool, and an explanation that “in the context of psychology and mental health, GenAI is beginning to be applied to various professional activities.” Participants then answered qualitative and quantitative questions assessing their attitudes toward GenAI technologies. Given the novelty of GenAI, participants were provided with the option to select a ‘not applicable/unable to answer’ response for survey items. This decision was made to avoid forcing participants to provide answers to items they felt were irrelevant or outside their experience.

### 3.3. Participants

Eligibility criteria included individuals who were practicing or trainee psychology professionals currently working within the NHS in the UK. Eligible professions included those listed in the UK Psychology Professions Workforce Census (NHS Benchmarking Network, 2023). Participants were recruited via social media, psychology conferences, and snowball recruitment. The target sample was 200, which is in line with conventional thresholds for SEM (Kline, 2023), and similar research (Kleine et al., 2023; Jussupow et al., 2022).

### 3.4. Measures

#### 3.4.1. Frequency of GenAI Usage

Frequency of use was measured in relation to participants’ personal life, clinical practice and other professional activities. A five-point Likert scale was used with response options of Often (Daily), Regularly (Weekly), Occasionally (Monthly), Rarely, and Never.

#### 3.4.2. Attitude Measures

For all questions, participants were prompted to consider their attitudes in relation to their clinical role, rather than other professional activities. Each item was rated on a five-point Likert scale (‘Strongly Agree’, ‘Agree’, ‘Neither Agree nor Disagree’, ‘Disagree’, ‘Strongly Disagree’). Across scales, wording was adapted to refer specifically to GenAI tools. The order of survey subscale items was randomised to reduce order effects (Bradburn et al., 2004). See Supplementary Section S1 for full scales and initial scale validation.

#### 3.4.3. Perceived Usefulness and Perceived Ease of Use

Perceived usefulness and perceived ease of use were each measured using six-item Likert scales (Davis, 1989), with higher scores indicating more positive perceptions. Both scales demonstrated high convergent, discriminant, and factorial validity in original validation studies, with excellent reliability (perceived usefulness α = 0.98; perceived ease of use α = 0.94; Davis, 1989). Both scales demonstrated excellent reliability in the present sample (perceived usefulness α = 0.956, CR = 0.957; perceived ease of use α = 0.930, CR = 0.931).

#### 3.4.4. Social Influence

Social influence was assessed using a three-item scale adapted from Van Der Ham et al. (2020), with higher scores indicating greater perceived social influence towards using GenAI technologies. A slight modification was made to item 2, whereby ‘supervisor’ was also specified, given the relevance of supervisory relationships for psychology professionals within NHS services. Van Der Ham et al. (2020) reported reliability of α = 0.664 for this scale in a healthcare sample. The scale demonstrated better reliability in the present sample (α = 0.796, CR = 0.815).

#### 3.4.5. Behavioural Intention to Adopt

Behavioural intention to adopt generative AI was measured using a three-item scale adapted from Venkatesh et al. (2003), with higher scores indicating greater behavioural intention. Items were adapted to specify that adoption was contingent on having ‘access and permission’, ensuring behavioural intention was not conflated with structural barriers to adoption within the NHS. Venkatesh et al. (2003) reported sufficient internal consistency in their original study (>0.70). The scale demonstrated excellent reliability in the present sample (α = 0.976, CR = 0.977).

#### 3.4.6. Facilitating Conditions

A facilitating conditions scale was initially included to capture individual and organisational conditions underpinning adoption. It was assessed using a four-item scale (Venkatesh et al., 2003) however the full scale showed poor reliability in the present sample (α = 0.505). Exploratory factor analysis highlighted that items FC3 and FC4 showed very low loadings (0.17 and 0.19), and so were removed. Conceptually, these items reflected organisational facilitating conditions, whereas FC1 and FC2 reflected individual ones. The retained two-item scale (FC1, FC2) demonstrated acceptable reliability (CR = 0.73, AVE = 0.58) but correlated highly with perceived ease of use (*r* = 0.79, exceeding its square root of AVE, 0.76), indicating a lack of discriminant validity. As the construct appeared to function as a proxy for perceived ease of use rather than a distinct factor, facilitating conditions were not retained in the final measurement model.

#### 3.4.7. Threats to Professional Capabilities

The threats to professional capabilities scale was adapted from existing measures used in healthcare contexts (Walter & Lopez, 2008; Jussupow et al., 2022). Items relating to control over clinical decision-making and patient care processes were taken from Walter and Lopez’s (2008) ‘perceived threat to professional autonomy’ scale while items relating to professional influence and ability to provide effective care were adapted from Jussupow et al.’s (2022) ‘threats to professional capabilities’ scale. Items referring specifically to medical decisions, allocation of scarce resources, ordering patient tests and general AI attitudes were excluded, given their lack of relevance for NHS psychology professionals and/or context-specific AI use.

The final scale comprised five items, with higher scores indicating higher perceived threat. Both Walter and Lopez (2008) and Jussupow et al. (2022) demonstrated good to excellent reliability for their respective scales (CR = 0.86–0.97 and CR = 0.91, respectively). The scale demonstrated excellent reliability in the present sample (α = 0.931, CR = 0.932).

#### 3.4.8. Self-Threat

Self-threat was assessed using a four-item ‘self-threat from AI’ scale (Jussupow et al., 2022). The scale was originally developed from Murtagh et al. (2012)’s ‘identity threat’ scale and subsequently adapted for the specific context of AI and healthcare professionals by Jussupow et al. (2022) (‘self-threat from AI’ scale). Items were adapted to be future- rather than present-focused (e.g., “undermines” was changed to “would undermine”) to assess behavioural intentions rather than current practice.

Murtagh et al. (2012) reported excellent reliability for this scale (α > 0.90), whilst Jussupow et al. (2022) reported good reliability (CR = 0.891). The scale demonstrated good reliability in the present sample (α = 0.895, CR = 0.898).

#### 3.4.9. Open Ended Qualitative Questions

Participants were also asked

(1) In what ways are you using GenAI tools (such as ChatGPT) within your clinical role?
(2) What are your reasons behind not using GenAI tools (such as ChatGPT) within your clinical role?

### 3.5. Ethics

Ethical approval was obtained from the University of Surrey Ethics Committee (Ref: 0844). The study adhered to the British Psychological Society (BPS) Code of Human Research Ethics (British Psychological Society, 2021).

### 3.6. Data Analysis

#### 3.6.1. Missing Data

Patterns of missingness were examined using the Missing Values Analysis procedure in SPSS (v31.0.0.0; IBM Corp., Armonk, NY, USA). Missing data were treated as missing at random (MAR) and handled using Full Information Maximum Likelihood (FIML) (Kline, 2023; Enders, 2022).

As a sensitivity analysis, given the higher rate of missing data on the social influence items, all structural models were additionally re-estimated across 20 multiply imputed datasets (reflecting the approximate percentage of missingness; White et al., 2011; Enders, 2022) to confirm that the findings were not dependent on the FIML approach.

#### 3.6.2. Quantitative Data Analysis Strategy

SPSS and jamovi (v2.7.6.0; The jamovi project, 2026, Sydney, Australia) were used to analyse data, with structural models estimated using the semlj module (v1.2.5) in jamovi, which is based on the lavaan package (v0.6.19; Rosseel, 2012). The multiple imputation sensitivity analysis was conducted in R (v4.6.1; R Core Team, 2026; R Foundation for Statistical Computing, Vienna, Austria) using the mice package (v3.19.0; van Buuren & Groothuis-Oudshoorn, 2011) and lavaan (v0.7.2; Rosseel, 2012).

To ensure data were suitable for analysis, all variables were assessed for univariate normality. Skew (–0.9 to +1.2) and Kurtosis (–1.2 to +1.8) scores fell within the acceptable thresholds (West et al., 1995). A two-step structural equation modelling (SEM) approach was employed (Anderson & Gerbing, 1988). First, confirmatory factor analysis (CFA) was conducted to verify the reliability and validity of latent constructs. Internal consistency was assessed using Composite Reliability (CR), convergent validity using Average Variance Extracted (AVE) and Discriminant validity using the Fornell–Larcker criterion. Items with standardised factor loadings below 0.50 were considered for removal (Bagozzi & Yi, 1988). Model fit was evaluated using CFI, TLI, RMSEA and SRMR. Conventional thresholds were used to determine acceptable fit (CFI/TLI ≥ 0.90, RMSEA/SRMR ≤ 0.08; Hair et al., 2019). Following confirmation of an acceptable measurement model, the hypothesised structural relationships were tested using SEM. In addition to the primary nested models outlined in Figure 1, a combined model including both identity threat constructs simultaneously was estimated to examine their relative contribution to behavioural intention. Bootstrapping with 5000 resamples was used and confidence intervals are reported for both direct and indirect effects.

#### 3.6.3. Qualitative Data Analysis Strategy

The qualitative data was analysed by the lead researcher using content analysis (H.-F. Hsieh & Shannon, 2005) and reviewed by co-authors. For Question 1, a conventional content analysis was conducted, with codes defined inductively, whereas responses for question 2 were analysed using a deductive approach using TAM as a framework. Responses were coded at the level of meaning units, so a single response could contribute multiple codes. Codes were organised into subcategories and categories. To avoid inflating prevalence, counts were deduplicated at each level of the hierarchy, such that a participant contributes at most once to any given subcategory or category regardless of how many codes they contributed to it. Reported n values therefore represent the number of participants referencing each category or subcategory. Because a participant could reference more than one subcategory within a category, subcategory counts do not sum to category totals.

## 4. Results

### 4.1. Extent and Patterns of Missing Data

Across the 32 survey items, most variables had very low levels of missingness (<5%), which is not expected to bias results (Kline, 2023). Exceptions included items within the Social Influence scale with missingness ranging from 17.4–24.4%. This high rate of missingness is likely attributable to the provision of a ‘not applicable’ response option (as described in the procedure), given that some psychology professionals may work independently or without direct supervisory structures, rendering peer or supervisor influence less relevant, or that norms around GenAI use had not yet formed within teams. Little’s MCAR test was not significant (χ^2^ = 589.6, *p* = 0.338) indicating that the hypothesis that data were Missing Completely at Random could not be rejected. Data were handled under the assumption of MAR using FIML.

### 4.2. Sample Characteristics

**Figure 2:**
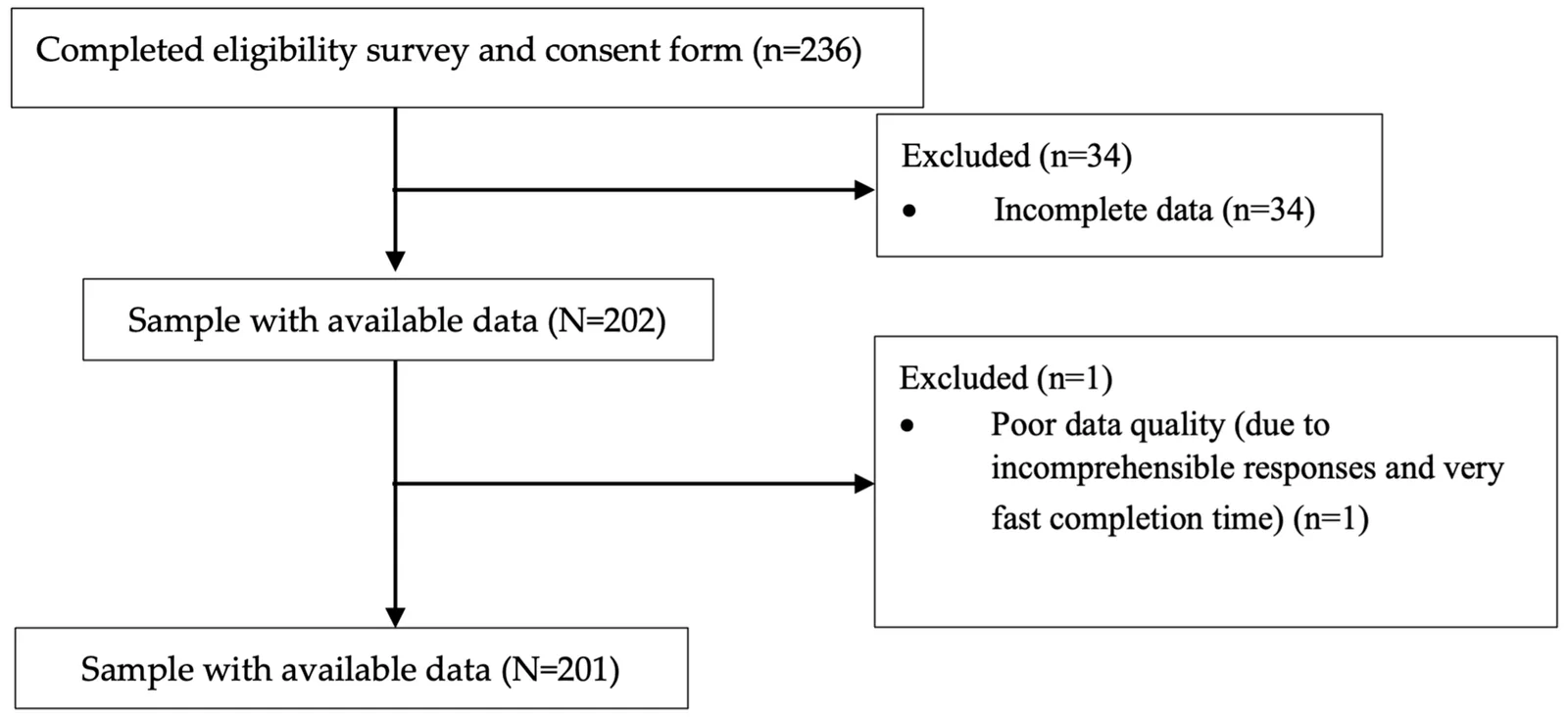
Attrition Rates.

A final sample of 201 participants was used for data analysis (see Figure 2 for attrition rates). The median time taken to complete the survey was 7 min and 45 s.

The majority of participants identified as female (82.1%) and White/White British (79.6%). Ages ranged from 18 to 78 years (*M* = 32.3), with an average of around seven years of clinical experience. See Table 1 for full demographic characteristics. Across professions, 71 participants (35%) were qualified staff. Most participants were psychologists (65.67%) working in adult community mental health services (26.9%). See Table 1 for full sample characteristics.

**Table 1:** Demographic and Professional Characteristics of Participants.

Sample Characteristic	Value	Qualified	Trainee	Unspecified	Total
Age (years), mean (*SD*)	32.3 (8.0)				
Gender (Female), *n* (%)	165 (82.1)				
Years of clinical experience (years), mean (*SD*)	6.9 (6.2)				
Ethnicity, *n* (%)					
Asian/Asian British	11 (5.5)				
Black/African/Caribbean/Black British	7 (3.5)				
Mixed/multiple ethnic groups	12 (6.0)				
Other ethnic group	9 (4.5)				
White/White British	160 (79.6)				
Prefer not to say	1 (0.5)				
Profession					
Psychologist		50	81	1	132
Clinical psychologist		44	80	1	125
Counselling psychologist		4			4
Forensic psychologist		1			1
Health psychologist			1		1
Other (please list)		1			1
Psychological Therapist		6	9		15
Cognitive Behavioural Therapist		4	8		12
Counsellor		1			1
Family and Systemic Psychotherapists		1			1
Psychological therapist (other)			1		1
Psychological Practitioner		7	6		13
Children Wellbeing Practitioner		1			1
Mental Health and Wellbeing Practitioner		1	1		2
Psychological Wellbeing Practitioner		4	4		8
Psychological practitioner (other)		1	1		2
Assistant or Associate Psychologist		1	6	13	20
Assistant Psychologist		1	3	13	17
Clinical Associate in Psychology			3		3
Other		7	14		21
Service context					
Adult inpatient mental health services					5
Adult community mental health services					54
Children and young people’s inpatient mental health services					1
Children and young people’s community mental health services					36
NHS Talking Therapies for anxiety and depression					24
Children and young people’s acute healthcare services					5
Adult acute healthcare services					27
Other					38
Prefer not to say/Not applicable					11

Most participants (98. 5%, *n* = 198) reported being familiar with the term "GenAI/ChatGPT". Usage was substantially higher in participants' personal lives than in clinically relevant activities (see Table 2).

**Table 2:** Participants’ frequency of usage of GenAI across different contexts.

Frequency	Personal		Clinical Role		Other Professional Contexts	
	n	%	n	%	n	%
Often (Daily)	25	12.4	12	6	19	9.5
Regularly (Weekly)	69	34.3	30	14.9	41	20.4
Occasionally (Monthly)	45	22.4	35	17.4	62	30.8
Rarely	34	16.9	40	19.9	32	15.9
Never	28	13.9	83	41.3	43	21.4

### 4.3. Measurement Model

Table 3 presents the means, standard deviations, reliability, and convergent validity statistics for all study constructs. *N* values represent participants with complete data for all items within each composite.

The measurement model demonstrated acceptable fit: χ^2^ (309) = 616, *p* < 0.001, CFI = 0.944, TLI = 0.936, RMSEA = 0.070 (95% CI [0.062, 0.078]), SRMR = 0.064. All standardised loadings exceeded the recommended 0.60 threshold (Bagozzi & Yi, 1988). All constructs showed acceptable internal consistency (α > 0.70; CR > 0.70), and convergent validity (AVE > 0.50).

Discriminant validity was supported by the Fornell–Larcker criterion, with each construct’s square-root AVE exceeding its correlations with all other constructs. All heterotrait–monotrait (HTMT) ratios fell below the conservative 0.85 threshold, with the exception of perceived usefulness and behavioural intention (0.88), which remained below the more liberal 0.90 threshold (Henseler et al., 2015). The two identity threat constructs were empirically distinguishable (HTMT = 0.77), supporting their treatment as distinct.

### 4.4. Structural Model Findings

#### 4.4.1. Model 1—Baseline Extended TAM

**Figure 3:**
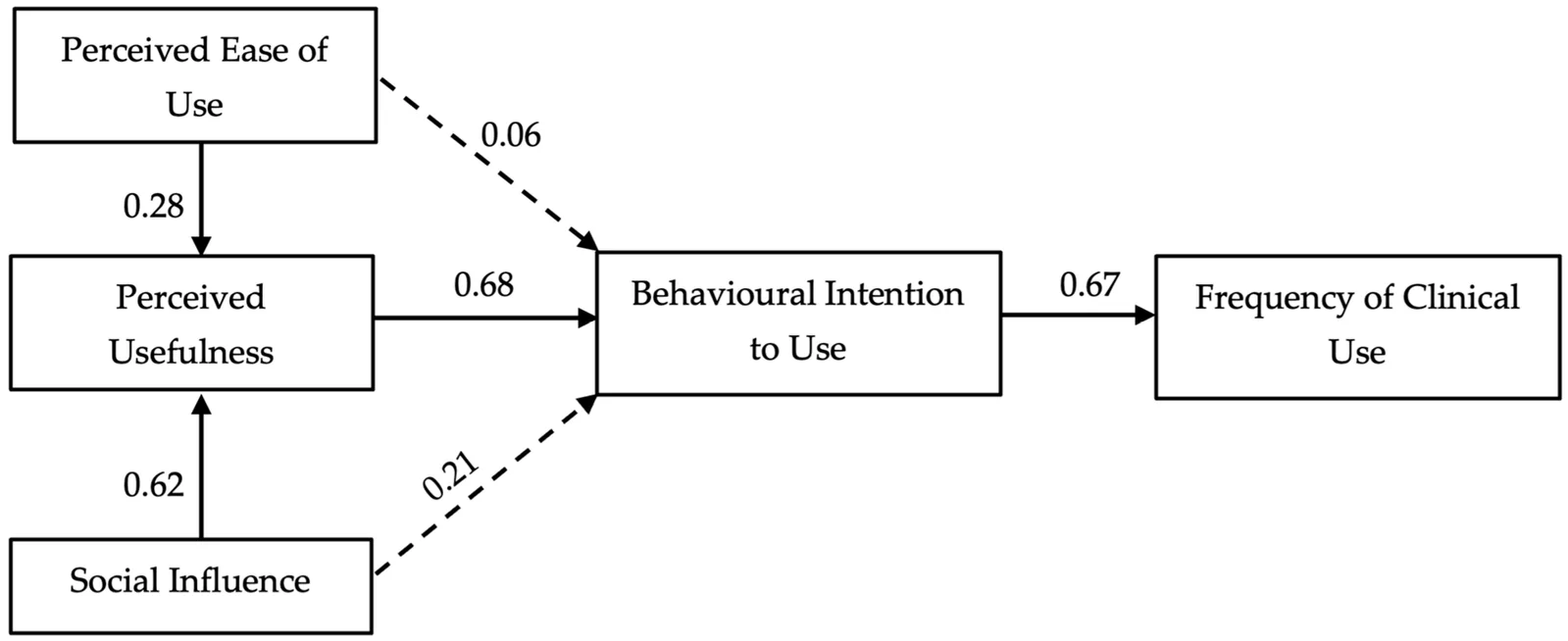
Model 1 pathway diagram. **Note.** Solid lines represent reliable effects (95% CI excludes zero); dashed lines represent effects that were not reliable (95% CI includes zero).

Model 1 demonstrated acceptable fit: χ^2^ (339) = 693, *p* < 0.001, CFI = 0.936, TLI = 0.929, RMSEA = 0.072 (95% CI [0.064, 0.080]), SRMR = 0.079. Model 1 explained 78.3% of the variance in behavioural intention, 45.1% of the variance in frequency of clinical use, and 63% of the variance in perceived usefulness. The standardised path estimates for Model 1 are shown in Figure 3.

Perceived usefulness exhibited a strong, reliable, direct association with behavioural intention (β = 0.68, 95% CI [0.42, 0.93]), which extended to frequency of clinical use (β = 0.45, 95% CI [0.28, 0.63]). Neither perceived ease of use (β = 0.06, 95% CI [−0.10, 0.22]), nor social influence (β = 0.21, 95% CI [−0.14, 0.56]), had a statistically reliable direct association with behavioural intention.

Social influence exerted a reliable indirect association with behavioural intention through perceived usefulness (β = 0.42, 95% CI [0.28, 0.56]), which extended to frequency of clinical use (β = 0.28, 95% CI [0.18, 0.38]). The indirect association of perceived ease of use with behavioural intention through perceived usefulness was positive but not statistically reliable (β = 0.19, 95% CI [−0.03, 0.40]).

#### 4.4.2. Model 2—Extended TAM + Threats to Professional Capabilities

**Figure 4:**
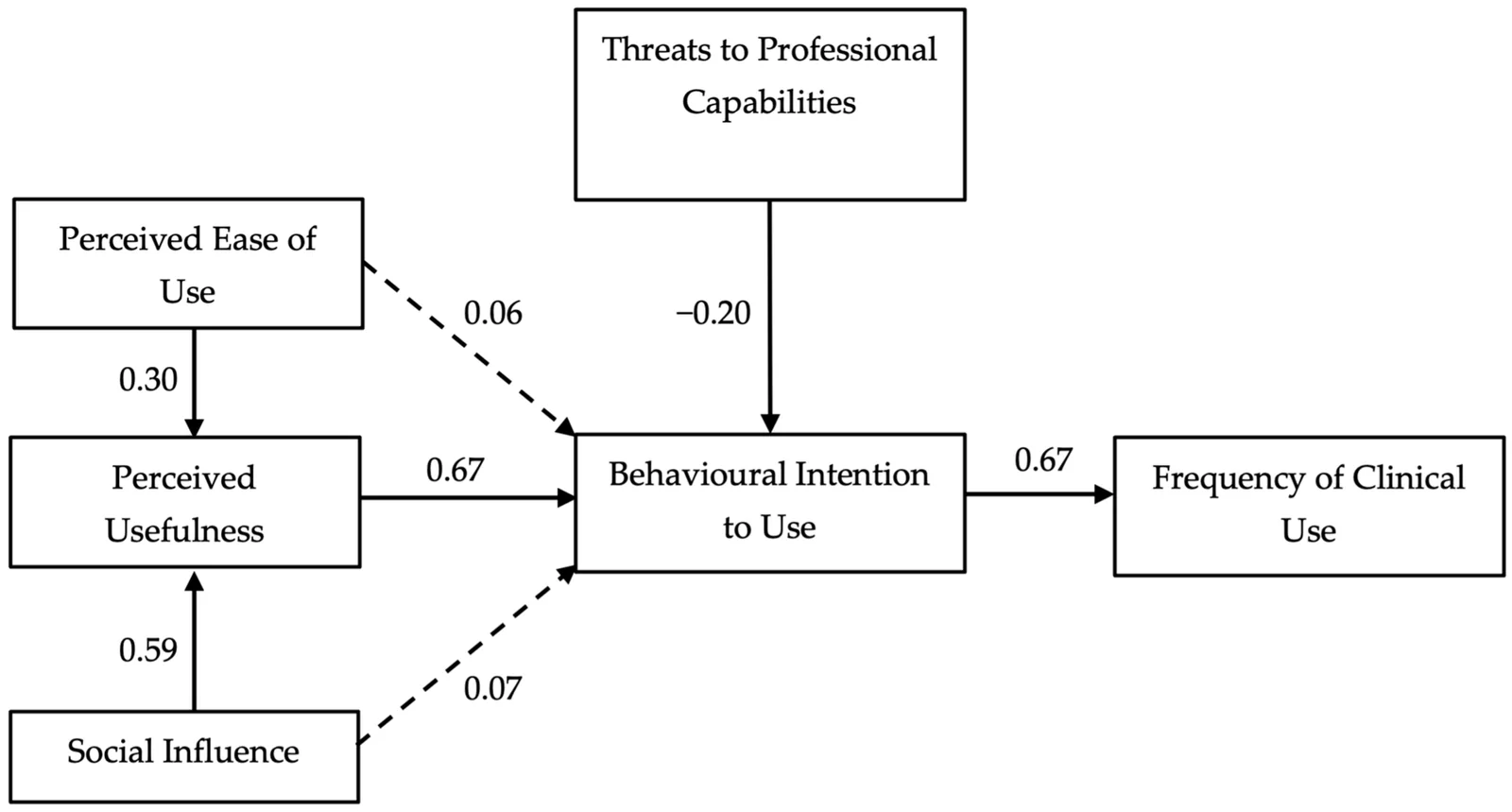
Model 2 pathway diagram. **Note.** Solid lines represent reliable effects (95% CI excludes zero); dashed lines represent effects that were not reliable (95% CI includes zero).

Adding threats to professional capabilities as an additional predictor produced a significant improvement in fit over Model 1, Δχ^2^ (1) = 13, *p* < 0.001, with acceptable overall fit: χ^2^ (338) = 680, *p* < 0.001, CFI = 0.938, TLI = 0.931, RMSEA = 0.071 (95% CI [0.063, 0.079]), SRMR = 0.076. Model 2 explained 78.7% of the variance in behavioural intention, 44.4% of the variance in frequency of clinical use, and 61.0% of the variance in perceived usefulness. The standardised path estimates for Model 2 are shown in Figure 4.

Threats to professional capabilities reliably and negatively associated with behavioural intention (β = −0.20, 95% CI [−0.32, −0.08]), which extended to frequency of clinical use (β = −0.14, 95% CI [–0.21, −0.06]). Perceived usefulness showed the strongest direct association with behavioural intention (β = 0.67, 95% CI [0.53, 0.81]), while neither perceived ease of use (β = 0.06, 95% CI [−0.04, 0.15]) nor social influence (β = 0.07, 95% CI [−0.10, 0.24]) showed a reliable direct association.

Indirect associations through perceived usefulness were reliable for both perceived ease of use (β = 0.20, 95% CI [0.04, 0.36]) and social influence (β = 0.39, 95% CI [0.25, 0.54]), each extending to frequency of clinical use (perceived ease of use: β = 0.13, 95% CI [0.03, 0.24]; social influence: β = 0.26, 95% CI [0.16, 0.37]).

#### 4.4.3. Model 3—Extended TAM + Self-Threat

**Figure 5:**
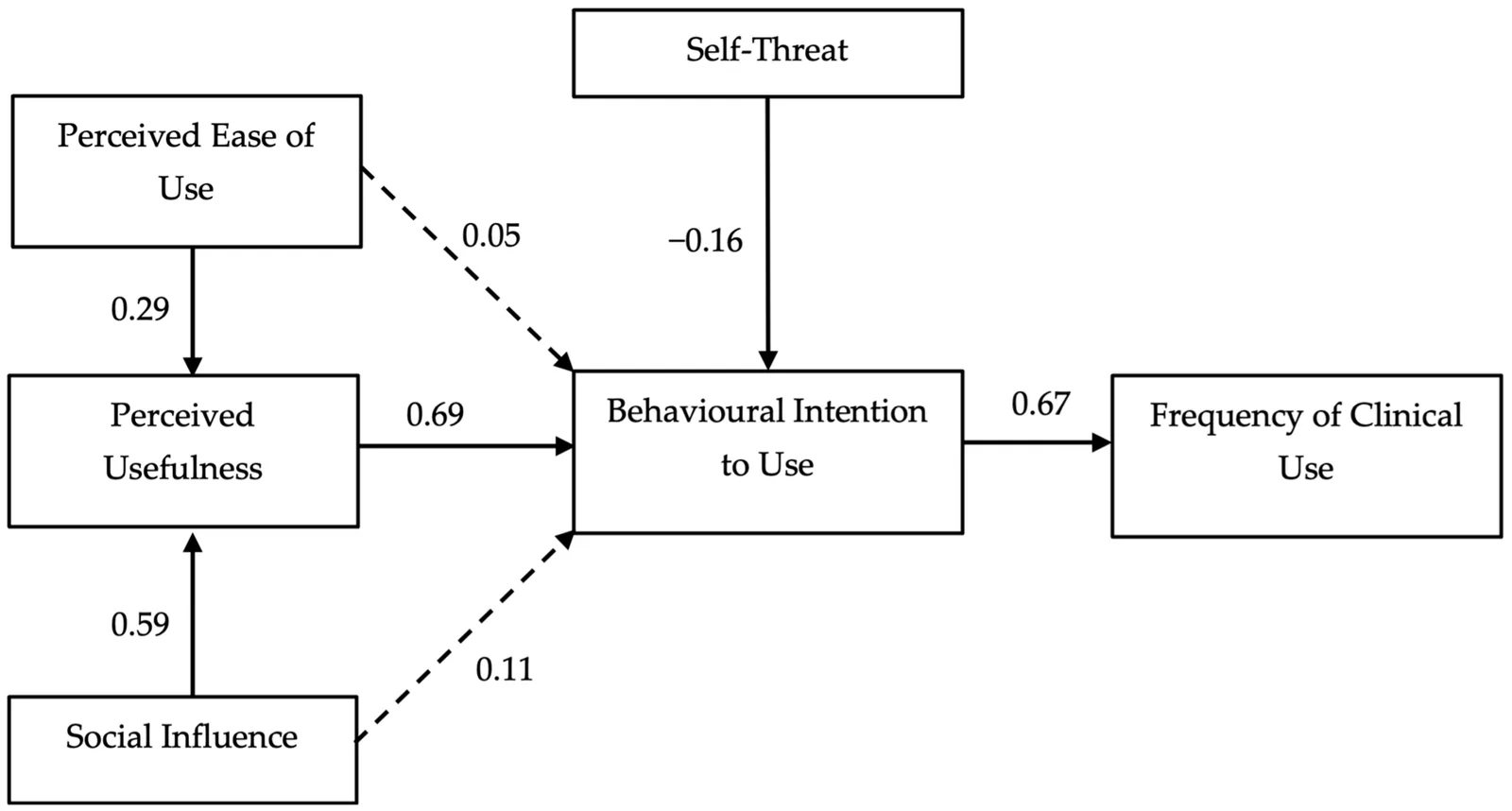
Model 3 pathway diagram. **Note.** Solid lines represent reliable effects (95% CI excludes zero); dashed lines represent effects that were not reliable (95% CI includes zero).

Adding self-threat as an additional predictor produced a significant improvement in fit over Model 1, Δχ^2^ (1) = 9, *p* = 0.003, with acceptable overall fit: χ^2^ (338) = 684, *p* < 0.001, CFI = 0.938, TLI = 0.931, RMSEA = 0.071 (95% CI [0.064, 0.079]), SRMR = 0.077. Model 3 explained 78.7% of the variance in behavioural intention, 44.6% of the variance in frequency of clinical use, and 61.5% of the variance in perceived usefulness. The standardised path estimates for Model 3 are shown in Figure 5.

Self-threat was reliably and negatively associated with behavioural intention (β = −0.16, 95% CI [−0.27, −0.05]), which extended to frequency of clinical use (β = −0.11, 95% CI [−0.18, −0.03]). As in the other models, perceived usefulness was the dominant correlate of behavioural intention (β = 0.69, 95% CI [0.53, 0.84]), and neither perceived ease of use (β = 0.05, 95% CI [−0.06, 0.15]) nor social influence (β = 0.11, 95% CI [−0.10, 0.32]) showed a reliable direct association.

Indirect associations through perceived usefulness were again reliable for perceived ease of use (β = 0.20, 95% CI [0.02, 0.38]) and social influence (β = 0.41, 95% CI [0.27, 0.55]), both extending to frequency of clinical use (perceived ease of use: β = 0.13, 95% CI [0.01, 0.25]; social influence: β = 0.27, 95% CI [0.17, 0.37]).

A summary of model fit statistics and comparisons across all three models is presented in Table 4.

**Table 4:** Model comparison table.

Model	Description	χ^2^ (*df*)	Δχ^2^ (*df*)	*p* Value	CFI	RMSEA	SRMR	*R*^2^ BI
Model 1	Extended TAM	693 (339)			0.936	0.072	0.079	0.783
Model 2	TAM + TPC	680 (338)	13 (1)	<0.001	0.938	0.071	0.076	0.787
Model 3	TAM + ST	684 (338)	9 (1)	0.003	0.938	0.071	0.077	0.787

**Note.** Δχ^2^ values reflect comparison against Model 1. TPC = Threats to Professional Capabilities; ST = Self-Threat; BI = Behavioural Intention to Use; CFI = Comparative Fit Index; RMSEA = Root Mean Square Error of Approximation; SRMR = Standardised Root Mean Square Residual.

#### 4.4.4. Combined Model

To determine whether threats to professional capabilities and self-threat each contributed to behavioural intention independently of one another, both were entered simultaneously in a combined model, which demonstrated acceptable fit (χ^2^(337) = 680, CFI = 0.938, TLI = 0.931, RMSEA = 0.071, SRMR = 0.076). Neither identity threat construct was reliably associated with behavioural intention (TPC β = −0.16, 95% CI [−0.35, 0.03]; ST β = −0.06, 95% CI [−0.22, 0.10]), and neither showed a reliable indirect association with frequency of clinical use. Given the strong association between the two constructs (HTMT = 0.77), the combined model has limited capacity to partition variance between them.

See Supplementary Section S4 for full model pathway coefficients and confidence intervals.

#### 4.4.5. Sensitivity Analysis

A multiple-imputation sensitivity analysis confirmed that the social influence findings were stable across methods of handling missing data with both direct and indirect effects remaining comparable in magnitude across all models (see Supplementary Section S5).

### 4.5. Content Analysis

Full content analysis table and additional supportive quotes are summarised in Supplementary Section S2 for use cases and Supplementary Section S3 for reasons for non-use.

#### 4.5.1. Current Use Cases

For current use cases, four primary categories were identified (*n* = 115 participants; see Table 5). Participants most frequently discussed using GenAI for Indirect Clinically Relevant Support (*n* = 69). This included activities such as looking up clinically relevant information, drafting letters and emails or generating session notes.

Using GenAI for Direct Clinically Relevant Support was also widely reported (*n* = 58). This included treatment and session planning, resource and material generation, and psychological formulation support. One participant reflected, “I use ChatGPT very frequently to help me with planning for sessions. This could be coming up with session plans or just brainstorming ideas”.

Participants also described using GenAI for Professional Learning, Reflection and Development (*n* = 37) that was related to their role but not directly tied to clinical work. This included using GenAI to develop their clinical skills more broadly, support with teaching and training, and general idea generation.

Research, Audit, and Service Development was the least frequently reported category (*n* = 10) and included use cases around support with data analysis and generating ideas for service development.

#### 4.5.2. Reasons for Non-Use

Eight primary categories were identified when considering reasons not to use AI (*n* = 146 participants; see Table 6). Ethical Concerns (*n* = 70) were the most frequently cited reasons for non-use, with concerns primarily relating to confidentiality and data protection. Some participants also cited general moral discomfort around using tools or concerns about the environmental impact of using GenAI.

Threat to professional identity was the joint second most prevalent reason for non-use (*n* = 36). Psychology professionals were primarily concerned that reliance on GenAI could reduce opportunities to practice and develop core clinical skills, including critical thinking, clinical judgement, reflective writing and engagement and problem-solving. They also worried that automating tasks such as reports and notes could diminish reflective engagement with clients and, over time, foster dependence and erode existing skills. As one participant reflected, “I feel like [using GenAI] might lead to a skill reduction in that particular area”. Participants also expressed concerns about how GenAI might threaten the relational and human nature of clinical work. As one participant reflected, “I believe the relational part of our work is the most important, and AI threatens this.” Finally, participants expressed concerns about how using GenAI might undermine their credibility or authenticity. This was often captured by participants feeling that using GenAI would feel like cheating. As one participant reflected, “[Using GenAI] would not feel like my authentic voice or my own work”.

Lack of trust was also cited as a prominent reason for non-use (*n* = 36), with many participants citing experiences of GenAI producing inaccurate or fabricated outputs. Lack of trust was also linked to the lack of transparency in how outputs were produced.

Lack of experience, knowledge and familiarity (*n* = 34) and lack of facilitating conditions (*n* = 31), including policy restrictions and governance barriers, were also frequent in responses. Some participants cited a perceived lack of usefulness (*n* = 26) or social influence (*n* = 6), and some simply reported general non-specific discomfort related to AI use (*n* = 11).

## 5. Discussion

### 5.1. Principal Findings

The present study set out to better understand the factors influencing psychology professionals’ adoption of GenAI technologies into their clinical roles. This was assessed using three extended TAM models which additionally incorporated identity threat variables. All models demonstrated acceptable fit and accounted for a large proportion of variance in behavioural intention (*R*^2^ = 0.78) and a moderate proportion in self-reported frequency of clinical use (*R*^2^ = 0.44–0.45). Perceived usefulness emerged as the dominant correlate of behavioural intention across models, whilst perceived ease of use and social influence both only operated indirectly through perceived usefulness. Although perceived usefulness remained the strongest correlate across models, both threats to professional capabilities and self-threat were reliably negatively associated with behavioural intention when considered separately, and both extended to frequency of clinical use.

### 5.2. Identity Threat Findings

The study’s findings have important implications for understanding how professional identity threat may impact the adoption of generative AI technologies within NHS psychological practice. Identity threat persisted as a correlate of behavioural intention while accounting for perceived usefulness, ease of use and social influence, consistent with H4 and H5. This represented a distinct pathway from how perceived ease of use and social influence operated which were both associated only indirectly through perceived usefulness. These findings extend research by Walter and Lopez (2008) and Jussupow et al. (2022) beyond medical professionals and provide a useful extension of the TAM within this adoption context as identity threat captures an appraisal of what the technology may mean for psychology professionals in terms of their autonomy, capabilities and sense of self that is not fully captured by perceived usefulness. This distinction presents the possibility for GenAI to be perceived as both useful and threatening simultaneously. The incremental variance attributable to identity threat was nonetheless small, increasing explained variance in behavioural intention by approximately 0.4% (*R*^2^ = 0.783 to 0.787). We therefore position identity threat not as the principal barrier to adoption, but as one that operates independently of the constructs implementation efforts often target. Because the two threat constructs were strongly related (HTMT = 0.77), they were modelled separately in the primary analyses. A supplementary model entering both simultaneously found neither to be reliably associated with behavioural intention, which is understandable given that the constructs are highly overlapping predictors competing for shared variance. This should not be read as evidence that identity threat is unimportant, since each construct was reliably associated with intention when entered separately. However, it does suggest that the two appraisals are not empirically separable with the present measures and sample, and that identity threat may be better treated as a single higher-order appraisal until instruments capable of distinguishing its role-based and self-related components are available.

The qualitative findings provide a further contextual lens by which identity threat may be experienced by psychology professionals within clinical practice. Participants’ most tangible concerns related to deskilling, in that use of GenAI might lead to a reduction in one’s own core skills such as clinical thinking ability or reflective capacity. Concerns of this kind may also represent plausible appraisals of genuine trade-offs rather than identity-protective responses, given emerging evidence from medical research that real-world exposure to AI leads to reduced engagement in diagnostic reasoning (Budzyń et al., 2025). Similar concerns are being raised for psychology professionals including the risk of AI eroding one’s ability to reflect on and justify clinical thinking (van Zyl, 2026).

Beyond this, participants also raised concerns about professional authenticity, expressed both as a sense of “cheating” and as doubt about whether AI-assisted work reflected their own voice. The feeling of cheating may partly reflect the instrumental risk that clinicians perceived as dependent on AI may be judged less competent than their peers (Yang et al., 2025). However, authenticity may also involve a more internal tension concerning whether AI-assisted work continues to feel genuinely one’s own. For psychology professionals, professional identity develops through increasing integration of the personal and professional self and authenticity is itself valued within professional development (Schubert et al., 2023). GenAI-generated material that does not reflect a clinician’s own language or reasoning may therefore create incongruence between their professional identity and the work produced in their name (Manzouri, 2026).

Related to the aforementioned concerns, participants’ identity-related barriers to use also centred on whether GenAI might diminish the human and relational processes underpinning clinical practice. This concern reflects something distinctive about psychological work, which is constituted not only by the production of clinically appropriate material but by the interpersonal processes through which it is developed and communicated, and in which the therapeutic relationship is among the most consistent predictors of outcomes (Flückiger et al., 2018). Föyen et al. (2025) offer some related evidence for this, finding that psychological advice believed to originate from a human was strongly preferred even when AI-generated advice was rated as more empathic and competent. This suggests that the value attributed to clinical practice may depend partly on its perceived human origin rather than solely on the quality of its content. A key question for future research is whether the concerns described here reflect genuine risks to delivering good quality psychological care, identity-protective responses to a technology that would in fact be beneficial, or a boundary between uses that support clinical work and those that undermine it. Establishing this will require research into how GenAI use affects the capabilities, authorship and relational processes participants described, and into how these concerns relate to one another.

### 5.3. Core TAM Findings

Across models, perceived usefulness emerged as the strongest correlate of behavioural intention, consistent with H1, broader technology acceptance literature (Venkatesh et al., 2003; Davis, 1989; King & He, 2006), and literature specifically relating to psychology professionals (Wagner & Schwind, 2025; Kleine et al., 2023). Qualitative findings provide contextual insight into what perceived usefulness may currently look like among participants already using GenAI, with respondents frequently describing use cases related to administrative and documentation tasks, supporting access to clinical information, generating clinical resources, and assisting with treatment planning and formulation. Whilst specific benefits of using GenAI within directly clinical activities are still largely unknown, emerging healthcare evidence has demonstrated the potential benefits of GenAI-assisted documentation in reducing administrative burden and cognitive workload (Olson et al., 2025; Shah et al., 2025).

Within our structural models, social influence did not exert a reliable direct association with behavioural intention but was consistently associated with intention through perceived usefulness, partially supporting H3. This implies that social influence shaped adoption intention primarily by signalling a technology’s usefulness rather than by operating as an independent driver of intention, consistent with TAM2 (Venkatesh & Davis, 2000; Schepers & Wetzels, 2007). Social influence has long been central to implementation strategy (Rogers, 2003), often mobilised through clinical champions, though evidence for their effectiveness in healthcare is mixed (Pettersen et al., 2024; Santos et al., 2022; Wood et al., 2020). The present findings suggest that social endorsement may be most influential when colleagues and supervisors demonstrate the concrete clinical value of a technology, rather than through normative pressure alone. Moreover, because identity threat was associated with behavioural intention independently of perceived usefulness, demonstrating the usefulness of GenAI may not be sufficient where identity-related concerns remain unaddressed.

Perceived ease of use did not exert a reliable direct association with behavioural intention in any model, contrary to H2. Its indirect association through perceived usefulness was reliable in the models incorporating identity threat (Models 2 and 3). This pattern partially supports H2 and is somewhat consistent with TAM2 (Venkatesh & Davis, 2000) and prior research (King & He, 2006), in which ease of use influences adoption primarily by shaping perceptions of usefulness rather than acting as an independent driver. The absence of a reliable direct effect mirrors Kleine et al. (2023), who suggested that in mental health contexts the intuitive appeal of highly user-friendly AI tools may sit in tension with the complex, high-stakes nature of clinical work. It may be the case, therefore, that for some use cases, such as those which are less clinically consequential, ease of use is more valued, however further research will be needed to fully understand this.

Interestingly, Participants on average rated GenAI as easy to use (*M* = 3.8), yet lack of experience and familiarity was among the frequently cited reasons for non-use. This apparent tension may reflect the distinction between technical usability and clinical proficiency. A clinician may find a conversational GenAI interface straightforward to operate while remaining uncertain about appropriate clinical applications, what information can safely be entered, whether organisational policy permits its use, or how its outputs should be verified. In this way, while a technology might be easy to use, it might be very difficult to understand its reliability, accuracy and explainability thereby inhibiting its usage in clinical contexts. This points to a wider AI skills gap within healthcare that is less about technological usability and more about proficiency in understanding AI technologies and being able to critically evaluate outputs (Chatzichristos et al., 2025).

### 5.4. Barriers Beyond the Structural Model

Some of the most frequently reported reasons for non-use fell outside the constructs our structural models tested, including data protection concerns, lack of trust in system outputs, and facilitating conditions. These mirror the barriers identified across the wider healthcare literature, where data privacy is one of the most frequently reported obstacles to AI adoption, alongside data quality, accuracy, trust, and regulations and governance (Abdelwanis et al., 2026). Barriers of this kind are not readily accommodated within the present study’s TAM framework, and Abdelwanis et al. (2026) argue that the field’s reliance on individual-level models limits its capacity to represent the organisational and environmental conditions shaping AI adoption in healthcare.

The facilitating conditions scale is instructive in this respect. It did not function coherently as a single construct as the items reflecting organisational conditions did not load, while the retained individual-level items were not empirically distinguishable from perceived ease of use. The construct was therefore not retained, with the consequence that organisational readiness was not modelled in this study. Yet organisational conditions were prominent in participants’ reasons for non-use, including limited approved access, unclear guidance, policy restrictions and uncertainty about information governance. A general-purpose instrument developed for organisationally provisioned technologies may map poorly onto GenAI in the NHS, where regulatory structures are complex, and individual familiarity with publicly available tools has developed faster than the approved infrastructure required for clinical use. Future measurement would benefit from separating individual from organisational readiness.

In relation to trust as a barrier, participants expressed concerns about inaccuracy, fabrication and lack of transparency in GenAI models. Interestingly, trust may not be entirely separable from professional identity. Ackerhans et al. (2025) found that greater trust in AI-based clinical decision support was associated with lower professional identity threat among physicians and medical students, and that increased explainability of outputs improved trust. Whether explainability or trust mediate the relationship between identity threat and adoption intention among psychology professionals would be a valuable question for future research.

### 5.5. Implications

The present study has implications for implementing GenAI into NHS psychological services. The finding that perceived usefulness was the dominant correlate of adoption intention suggests that implementation efforts may benefit from prioritising demonstrating tangible clinical benefits of GenAI technologies such as reducing administrative burden and freeing clinician time for direct clinical work (Olson et al., 2025; Shah et al., 2025). At the same time, identity threat findings highlight that simply considering usefulness might not be sufficient to drive adoption. Implementation strategies should focus on preserving clinician authorship and autonomy over how GenAI is introduced into clinical roles, and introducing tools that support rather than replace clinical reasoning, in order that clinicians are able to retain both their clinical capabilities and their sense of professional authenticity. Our social influence findings suggest that clinical champions and peer endorsement may be more effective when they demonstrate what a tool does for clinical work, rather than signalling that colleagues approve of it. While our findings do not point to technological ease of use as a primary target for implementation, lack of experience and knowledge was among the most frequently cited reasons for non-use. Training focused on operating these tools may therefore be insufficient. The findings suggest a need for evaluative competence, including which tasks are appropriate, what information may safely be entered, and how outputs should be verified. Finally, findings suggest that individual-level implementation efforts will have limited traction until the wider conditions of use are addressed, including data protection improvements, clear guidance and information governance, alongside improved accuracy and explainability of the tools themselves.

### 5.6. Limitations

Several limitations should be noted. First, due to the cross-sectional design, although our data support hypothesised directional relationships, we cannot claim causality (Maxwell & Cole, 2007). Second, the self-selecting recruitment may have introduced bias, attracting participants who were more accepting of, or familiar with, GenAI technologies (Bethlehem, 2010). Our findings, therefore, may better represent the views of early adopters who tend to put more value on perceived usefulness rather than social influence (Rogers, 2003). Third, sample limitations should be noted as our sample was made up of predominantly female, White British, and relatively young clinicians with a substantial proportion of clinical psychologists and trainees. Therefore, we were unable to explore the moderating effects of demographic and professional characteristics. Additionally, this limits the generalisability of the findings as identity-threat may present differently across career stages and professional groups. Future research should examine whether the relationships identified here are replicated among different practitioner groups.

Fourth, no validated scale exists to measure professional identity threats among psychology professionals. The present study adapted scales from medical contexts where professional recognition is closely tied to professional identity (Jussupow et al., 2022). This differs from psychological professionals, for whom professional identity is more influenced by personal identity rather than status or recognition (Alves & Gazzola, 2011; Schubert et al., 2023). Given that psychology professionals’ professional identities are closely intertwined with their personal identities (Schubert et al., 2023; Verling, 2014; Alves & Gazzola, 2011), self-threat was included as an additional identity threat variable given its theoretical link to professional identity within this context. Future research should aim to refine and validate a scale that captures the unique ways in which professional identity threats manifest for psychology professionals, including whether role-based and self-related components of identity threat are empirically distinguishable in this group.

Finally, the heterogeneity of GenAI as a technology means participants may have responded with different use cases in mind, ranging from administrative support to more clinically consequential uses such as treatment planning. This might have resulted in variability in the relationships between variables (Kleine et al., 2023). Future research should explore adoption intentions across various use cases to determine how identity-related mechanisms are present across contexts of use.

## 6. Conclusions

The present study offers an exploration of psychology professionals’ attitudes toward GenAI within NHS clinical practice. Our findings indicate first and foremost that perceived usefulness remains the dominant correlate of GenAI adoption within NHS psychological practice, whilst ease of use and social influence were associated with behavioural intention primarily through perceived usefulness. However, the picture is more complex within psychological practice, where GenAI may be perceived as simultaneously useful in terms of supporting both clinical and non-clinical activities, whilst also threatening the clinical capabilities, professional authenticity and relational foundations through which clinicians develop their identities. This all exists within an adoption context defined by complex organisational and regulatory structures. As GenAI becomes increasingly sophisticated, understanding how its integration affects the clinical competencies, professional roles and human-to-human relationships that have traditionally defined psychological practice will become increasingly important.

## Figures and Tables

**Table 3:** Descriptive Statistics, Reliability and Convergent Validity Statistics for Study Constructs.

Construct	N	Mean	StDev	α	CR	AVE	SQRT_AVE
PU	198	3.63	1.02	0.956	0.957	0.788	0.888
PEOU	189	3.80	0.86	0.930	0.931	0.693	0.832
SI	130	2.50	0.84	0.796	0.815	0.597	0.773
TPC	194	2.92	1.08	0.931	0.932	0.732	0.856
ST	197	2.55	1.06	0.895	0.898	0.688	0.829
BI	196	3.42	1.25	0.976	0.977	0.934	0.966

**Note.** PU = Perceived Usefulness; PEOU = Perceived Ease of Use; SI = Social Influence; TPC = Threat to Professional Capabilities; ST = Self-Threat; BI = Behavioural Intention to Use; AVE = Average Variance Extracted.

**Table 5:** Content Analysis Categories and Subcategories: Current Use Cases of Generative AI.

Category/Subcategory	*n*
A: Indirect Clinically Relevant Support	69
A1: Accessing Clinically Relevant Information	42
A2: Clinically Related Administration Support	43
B: Direct Clinically Relevant Support	58
B1: Treatment Planning	32
B2: Resource/Material Generation	26
B3: Psychological Formulation Support	15
C: Professional Learning, Reflection and Development	37
C1: Personal and Professional Development	21
C2: Support for Teaching/Training	11
C3: Idea Generation	5
D: Research, Audit, and Service Development	10
D1: Research, Audit and Service Development	10

**Note.** Categories are not mutually exclusive. *n* = number of participants referencing each category or subcategory. Total participants = 115. Full subcategory detail and illustrative quotes are provided in Supplementary Section S2.

**Table 6:** Content Analysis Categories and Subcategories: Reasons for Non-Use of Generative AI.

Category/Subcategory	*n*
A: Ethical Concerns	70
A1: Ethical and Confidentiality Concerns	66
A2: Broader Societal and Environmental Concerns	10
B: Threat to Professional Identity	36
B1: Threat to Professional Capabilities	21
B2: Relational and Human Nature of Clinical Work	11
B3: Threats to Professional Authenticity	12
C: Lack of Trust	36
C1: Inaccuracy/Fabrication	28
C2: Lack of Transparency	4
C3: Preference for Verified Human Sources	3
C4: General Distrust or Scepticism	6
D: Lack of Experience, Knowledge and Familiarity	34
D1: Lack of Time to Explore	4
D2: Lack of Training	2
D3: Low Confidence in Use	3
D4: Unfamiliarity	18
D5: Low Salience/Lack of Habit	13
E: Facilitating Conditions	31
E1: GDPR and Governance	12
E2: Lack of Access or Equipment	4
E3: Lack of Guidance or Support	6
E4: Policy/Trust Restrictions	13
F: Perceived Lack of Usefulness	26
F1: Lack of Perceived Usefulness	3
F2: No Identified Use-Case	6
F3: Perceived Irrelevance to Role	15
F4: Preference for Traditional Methods	4
G: Affective Attitudes	11
G1: Emotional Discomfort About Usage	11
H: Social Influence	6
H1: Fear of Judgement	4
H2: Lack of Social Norms	4

**Note.** Categories are not mutually exclusive. *n* = number of participants referencing each category or subcategory. Total participants = 146. Full subcategory detail and illustrative quotes are provided in Supplementary Section S3.

## Data Availability

The data presented in this study are available on request from the corresponding author.

## Supplementary Materials

The following supporting information can be downloaded at: https://www.mdpi.com/article/10.3390/bs16101827/s1, Supplementary Section S1: Survey Questionnaire and Scale Validation. Supplementary Sections S2 and S3: Content Analysis Categories, Subcategories and Example Quotes. Supplementary Section S4: Full Model Pathway Coefficients. Supplementary Section S5: Multiple Imputation Sensitivity Analysis Comparisons.
