# Supplementary material for: Identity Threat as a Barrier to NHS Psychology Professionals’ Adoption of Generative AI: A Technology Acceptance Model Study
Source: Behav Sci (Basel). 2026 Oct 6;16(10):1827. doi: 10.3390/bs16101827 (PMC13701167; doi:10.3390/bs16101827)
Supplement: Supplementary file 1 [file behavsci-16-01827-s001.zip › behavsci-4479966-supplementary.pdf]

Supplementary Section S1: Survey Questionnaire and Scale Validation

Table S1. Survey Items and Standardised Loadings by Scale

Scale and item	N	Standardised loadings
Perceived Usefulness		
PU1: Using Generative AI tools in my job would enable me to accomplish tasks more quickly	200	0.847
PU2: Using Generative AI tools would improve my job performance	198	0.889
PU3: Using Generative AI tools in my job would increase my productivity	199	0.879
PU4: Using Generative AI tools would enhance my effectiveness on the job	199	0.900
PU5: Using Generative AI tools would make it easier to do my job	199	0.896
PU6: I would find Generative AI tools useful in my job	199	0.913
Perceived ease of use		
PEOU1: Learning to operate Generative AI tools would be easy for me	200	0.870
PEOU2: I would find it easy to get Generative AI tools to do what I want it to do	196	0.836
PEOU3: My interaction with Generative AI tools would be clear and understandable	195	0.777
PEOU4: I would find Generative AI tools to be flexible to interact with	191	0.774
PEOU5: It would be easy for me to become skillful at using Generative AI tools	200	0.852
PEOU6: I would find Generative AI tools easy to use	198	0.876
Social influence		
SI1: Most of my patients would welcome me using Generative AI tools	161	0.660
SI2: My superior(s)/supervisor(s) think(s) that I should use Generative AI tools	152	0.765
SI3: Colleagues who are important to me think I should use Generative AI tools	166	0.878
Facilitating conditions[a]		
FC1: I have the resources necessary to use Generative AI tools[a]	197	0.680
FC2: I have the knowledge necessary to use the Generative AI tools[a]	201	0.820
FC3: Generative AI tools are not compatible with other systems I use[a]	177	0.174
FC4: A specific person (or group) is available for assistance with Generative AI tools[a]	167	0.191
Threat to Professional Capabilities		
TPC1: Using Generative AI tools may decrease clinicians' control over clinical decisions	199	0.815
TPC2: Using Generative AI tools may decrease clinicians' control over each step of the patient care process	198	0.867
TPC3: Using Generative AI tools may decrease my professional discretion over patient care decisions	200	0.811
TPC4: I fear that when using Generative AI tools, clinicians would have less influence on patient care	197	0.912
TPC5: I would find generative AI advantageous for my profession as a whole[a]	200	0.663
TPC6: I fear that when using Generative AI, clinicians would be less able to treat their patients well	199	0.868
Self-Threat		
ST1: Using Generative AI tools in my clinical role would undermine my sense of self-worth	201	0.871
ST2: Using Generative AI tools in my clinical role make me feel less competent	198	0.864
ST3: Using Generative AI tools in my clinical role would require changing who I am	200	0.729
ST4: Using Generative AI tools in my clinical role would make me feel less unique as a person	200	0.846
Behavioural Intention		
BI1: Assuming I have access and permission, I intend to use generative AI tools in my clinical role	196	0.991
BI2: Assuming I have access and permission, I predict I would use generative AI tools in my clinical role	199	0.929
BI3: Assuming I have access and permission, I plan to use generative AI tools in my clinical role	199	0.978

Note: [a]Not retained in the final measurement model (see Methods).

Supplementary Section S2: Content Analysis Categories, Subcategories and Example Quotes for Survey Question 1: Generative AI Current Use Cases

Table S2

Category A: Indirect Clinically Relevant Support (*n* = 69)

Participants describing use cases that are relevant to, but not directly related to, specific pieces of clinical work.

	**Examples**
**Subcategory A1: Accessing Clinically Relevant Information (*n* = 42)**<br>• Looking up evidence base (*n* = 19)<br>• Quick clinically relevant information lookup (*n* = 14)<br>• Evidence base summarisation (*n* = 12)<br>• Signposting (*n* = 3)	"For quick summaries of psychological concepts/theories, or research summaries"<br><br>"Summary of guidelines relating to a particular diagnosis."<br><br>"If I want to look up something about a particular condition etc but am short on time I might do a quick search on Chat GTP e.g. what is Structured Clinical Management or something along those lines"<br><br>"Collating local resources"
**Subcategory A2: Clinically related administration support (*n* = 43)**<br>• Professional communication (*n* = 17)<br>• Drafting clinical letters or reports (*n* = 12)	"To create templates for clinical documentation that I can personalise later"<br><br>"Rewording letters for clarity"

• Transcribing / generating session notes (*n* = 8)<br>• Summarising notes/information (*n* = 5)<br>• Creating templates for reports or documentation (*n* = 4)<br>• General information summarisation (*n* = 3)<br>• General writing support (*n* = 3)<br>• Organisational and productivity support (*n* = 2)	"Proof reading my administrative work"<br><br>"Transcribe and generate session notes"<br><br>"Wording of written communications mainly (always anonymised)"

Table S3

Category B: Direct Clinically Relevant Support (*n* = 58)

Participants describing use cases that are directly related to specific pieces of clinical work.

	**Examples**
**Subcategory B1: Treatment planning (*n* = 32)**<br>• Treatment or session planning (*n* = 16)<br>• Generating specific intervention ideas (*n* = 6)<br>• Group planning (*n* = 5)	"Generate ideas for sessions e.g. behavioural experiments, mindfulness exercises, sensory toolboxes"<br><br>"To generate creative and effective ideas for adapting sessions and/or homework for particular service user needs i.e. CBT

• Adapting interventions to client needs (*n* = 5)<br>• Creating client-facing therapeutic resources (*n* = 1)<br>• Generating specific intervention ideas (*n* = 1)	behavioural activation for individuals with ADHD"
**Subcategory B2: Resource/material generation (*n* = 26)**<br>• Resource/material generation (*n* = 30)<br>• Tailoring, simplifying or translating materials (*n* = 11)<br>• Creating tailored metaphors/stories for clinical work (*n* = 8)<br>• Generating handouts or worksheets (*n* = 5)<br>• Creating visuals or images for therapy or psychoeducation (*n* = 4)<br>• Using generative AI to create client-facing therapeutic resources (*n* = 2)	"To create metaphors to help explain clinical concepts- can make relevant to individual clients"<br><br>"For new techniques and to develop resources (for example I had a client with a special interest being disney so asked it to give me three disney themed breathing exercises that I then developed into my own resource)"<br><br>"To create text for patient information leaflets at an appropriate reading age"<br><br>"For summarising psychoeducation materials for example in the context of CFT and what the key information is that I should include in sharing as part of psychoeducation"

**Subcategory B3: Psychological Formulation Support (*n* = 15)**	"I have put in anonymous details to think about what I might be missing. When supervisor has not been available to ask questions about a case, model, formulation. to consider a more integrative approach"<br><br>"Check if I'm taking the correct course of action with a client or what to respond to a client when presenting with complex difficulties. Usually just double-checking if I'm doing the right thing"

Table S4

Category C: Professional Learning, Reflection and Development (*n* = 37)

Participants describing use cases relating to personal and professional learning and training tasks.

	**Examples**
**Subcategory C1: Personal and professional development (*n* = 21)**<br>Categories are not mutually exclusive. n = number of participants referencing each category or Subcategory. Because a participant could reference more than one Subcategory within a category, Subcategory	"I use it to help me with my training- eg to role play with me and demo Socratic questioning"<br><br>"I also use it to develop my understanding of psychological models/intervention"

counts do not sum to category totals. Total responses = 115. Full Subcategory detail and illustrative quotes are provided in the Supplementary Materials.<br>• Using AI to develop clinical knowledge/skills (*n* = 17)<br>• Reflective practice (*n* = 4)<br>• Delivering feedback (*n* = 3)	"I have found it useful for asking questions to prompt reflections"<br><br>"I have also used it to help with providing feedback to supervisees"
**Subcategory C2: Support for teaching/ training (*n* = 11)**	"Brainstorm ideas and contents for training sessions"<br><br>"Teaching sessions on a specific topic for a specific discipline (eg 30 minute teaching session). I have used AI to ensure I include the most relevant information in the timeframe that I have been given"
**Subcategory A3: Idea generation (*n* = 5)**<br>• Generating questions (*n* = 3)<br>• Generating ideas (*n* = 2)	"Bouncing off ideas"<br><br>"Generating questions"

Table S5

Category D: Research, Audit, and Service Development (*n* = 10)

Participants describing use cases relating to research, audit and service development.

**Category D: Research, Audit, and Service Development (*n* = 10)**	**Examples**
**Subcategory D1: Research, Audit and Service development 10**<br>• Data management and statistics (*n* = 3)<br>• Research support (*n* = 3)<br>• Service development ideas (*n* = 2)<br>• Support with research methods (*n* = 1)<br>• Audit support (*n* = 1)	"Helping to organise data management. Helping with statistical analysis"<br><br>"I've asked it to help me come up with ideas on how to improve the quality of referrals. I've asked it to help me think of ways to evidence the need for increased resource for the service and how to make a case for this"<br><br>"Recently in my team we used it to generate ideas around strategies to increase referrals to the service taking into account our limited staffing capacity"

Supplementary Section S3: Content Analysis Categories, Subcategories and Example Quotes for Survey Question 2: Reasons for Non-Use of Generative AI

Table S6

Category A: Ethical concerns (*n* = 70)

Perceptions that using AI may violate ethical or moral principles, including harm to society, the environment, data protection, or patient rights.

	**Examples**
**Subcategory A1: Ethical and Confidentiality Concerns (*n* = 66)**<br>• Confidentiality & Data Protection (*n* = 56)<br>• Ethical Discomfort/Moral Concerns (*n* = 15)<br>• Lack of patient consent/informed use (*n* = 3)	"I am worried that using Gen AI to do notes/write letters etc would compromise confidentiality for patients and staff."<br><br>"Unclear how data inputted is used in future."<br><br>"I worry that AI outputs could be harmful."<br><br>"Ethical concerns around the originality of its content which may be regurgitated from other sources."<br><br>"I think it doesn't sit right with me ethically as service users are not at all informed that this is something we do."

**Subcategory A2: Broader Societal and Environmental Concerns (*n* = 10)**<br>• Environmental Impact (*n* = 10)<br>• General societal concerns (*n* = 1)	"AI tools use an incredible amount of energy and resources. Their environmental impact is huge. I would never use them given how much energy they use."<br><br>"I am concerned about the climate cost of running large language models."<br><br>"I don't wish to use AI for social justice reasons and to be conscious of our impact on the world."

Table S7

Category B: Threat to Professional Identity (*n* = 36)

Fears that AI undermines core psychological skills, therapeutic relationships, or what it means to be a competent, authentic, human clinician.

	**Examples**
**Subcategory B1: Threat to professional capabilities (*n* = 21)**<br>• Deskilling/Over-Reliance (*n* = 16)<br>• Erosion of Reflective Practice (*n* = 3)	"Worry that this may eventually lead to me not learning how to do these things properly myself."

• Job-Loss Concerns (*n* = 3)	"I also think that notes and letters are an important part of the therapeutic process. I think that using gen AI to make these tasks quicker neglects these aspects of therapeutic work as you don't spend as long considering the person and formulating."<br><br>"I am hesitant to contribute to AI potentially replacing or threatening jobs or skills in the future."
**Subcategory B2: Relational and Human Nature of Clinical Work (*n* = 11)**<br>• Importance of Human Relationship (*n* = 8)<br>• Lack of Empathy/Emotional Understanding (*n* = 3)<br>• Threat to Therapeutic Alliance (*n* = 6)	"When I synthesise and formulate and summarise there is a human and emotional and relational element to that that wouldn't be replicated by AI."<br><br>"Therapy and psychological work is inherently human and relational, and AI lacks this."<br><br>"I can't imagine anything more invalidating than reading an incorrect, obviously AI-generated sentence in your assessment or therapy letter."

**Subcategory B3: Threats to Professional Authenticity (*n* = 12)**	"I think I feel like I should be able to do it without ChatGPT. It kind of feels like cheating somehow."

Table S8

Category C: Trust (*n* = 36)

Concerns about whether AI outputs are accurate, reliable, transparent, or epistemically trustworthy enough for clinical work.

	**Examples**
**Subcategory C1:** Inaccuracy/Fabrication (*n* = 28)	"Inaccurate. Often wrong. Prone to misinterpretation or to giving user-liked answers that are not the truth."<br><br>"I've used AI for some academic work and it's gotten things wrong, so I wouldn't trust it for clinical guidance."
**Subcategory C2:** Lack of Transparency (*n* = 4)	"Concerns about how AI systems learn and integrate information."<br><br>"Unsure of the provenance of some of the suggestions it can make."
**Subcategory C3:** Preference for Verified Human Sources (3)	"I would only trust the clinical judgement of a human being."

**Subcategory C4:** General Distrust or Scepticism (6)	"I don't trust it."<br><br>"[I] feel sceptical about it."

Table S9

Category D: Lack of Experience, Knowledge and Familiarity (*n* = 34)

Lack of familiarity, skill, confidence, or exposure to AI tools, including not knowing how or when one might use them.

	**Examples**
**Subcategory D1:** Lack of Time to Explore (*n* = 4)	"Having time to practice and try things out is also a barrier."
**Subcategory D2**: Lack of training (*n* = 2)	"Not feeling that I understand it enough."
**Subcategory D3:** Low Confidence in Use (*n* = 3)	"Not feeling confident in using it."
**Subcategory D4:** Unfamiliarity (*n* = 18)	"Still becoming aware of its functions."<br><br>"I'm not familiar with the technology yet and wouldn't know how to use it to get the best results."
**Subcategory D4:** Low Salience/ Lack of Habit (*N* = 13)	"I have never even considered it as an option until now. I don't use generative AI often in

	general so I just hadn't thought about using it in a clinical context."

Table S10

Category E: Facilitating Conditions (*n* = 31)

Structural or organisational factors that make AI use difficult or impossible, including policy restrictions, lack of access, governance barriers or unclear guidance.

	**Examples**
**Subcategory E1:** GDPR & Governance (*n* = 12)	"I'm unsure if there are rules around using it clinically."<br><br>"Would use more if not for GDPR."
**Subcategory E2:** Lack of Access or Equipment (*n* = 4)	"Access to ChatGPT blocked in NHS computers (when using NHS wifi)."<br><br>"Lack of facilities in NHS setting."
**Subcategory E3:** Lack of Guidance or Support (*n* = 6)	"There is a lack of guidelines on the safe and appropriate use of AI in clinical practice. "
**Subcategory E4:** Policy/Trust Restrictions (*n* = 13)	"I'm not sure whether this would be permitted by my trust"

Table S11

Category F: Perceived Usefulness (*n* = 26)

Beliefs about whether Gen AI is relevant or valuable to one's role

	**Examples**
**Subcategory F1:** Lack of perceived usefulness (*n* = 3)	"If I try and anonymise the information or simplify it, then the output I am going to get is also going to be simplified and less useful."<br><br>"Surface-level analysis."
**Subcategory F2:** No Identified Use Case (*n* = 6)	"Not sure what I would use it for."
**Subcategory F3:** Perceived Irrelevance to Role (*n* = 15)	"Even if I did think it was appropriate, I offer manualised and protocol-based treatments and so I don't feel AI can be of much use in terms of offering clinical guidance."<br><br>"I haven't found an area of my practice which I have thought could be streamlined or improved by using AI tools."

**Subcategory F4:** Preference for Traditional Methods (*n* = 4)	"Availability of alternative resources such as lecture notes, textbooks, journals, supervision, peers."

Table S12

Category G: Affective Attitudes (*n* = 11)

Emotional reactions, gut feelings, and intuitive discomfort toward using AI, independent of its actual performance or practical barriers.

	**Examples**
**Subcategory G1**: Emotional discomfort about usage (*n* = 11)	"In relation to possibly using AI to support with cases, it just generally feels wrong."<br><br>"I have deep reservations about it being used clinically."

Table S13

Category H: Social influence (*n* = 6)

Perceptions about how socially acceptable AI use is, including fear of judgment and social norms.

	**Examples**

**Subcategory H1:** Fear of judgement (*n* = 4)	"I also worry how others might view me for using it."<br><br>"Some people have very negative views about it."
**Subcategory H2:** Lack of social norms (*n* = 4)	"Not many people in my team use it."

Supplementary Section S4: Full Model Pathway Coefficients

Table S14. Standardised Path Estimates Across All Model Specifications

**Path**	**Model 1**	**Model 2 (+TPC)**	**Model 3 (+ST)**	**Combined (TPC+ST)**
**Direct on BI**				
PU → BI	0.68 [0.42, 0.93]	0.67 [0.53, 0.81]	0.69 [0.53, 0.84]	0.67 [0.54, 0.81]
PEOU → BI	0.06 [–0.10, 0.22]	0.06 [–0.04, 0.15]	0.05 [–0.06, 0.15]	0.05 [−0.05, 0.15]
SI → BI	0.21 [–0.14, 0.56]	0.07 [–0.10, 0.24]	0.11 [–0.10, 0.32]	0.07 [−0.10, 0.24]
TPC → BI	—	-0.20 [–0.32, –0.08]	—	−0.16 [−0.35, 0.03]
ST → BI	—	—	-0.16 [–0.27, –0.05]	−0.06 [−0.22, 0.10]
		Direct on PU/Freq		
PEOU → PU	0.28 [0.03, 0.52]	0.30 [0.08, 0.51]	0.29 [0.07, 0.52]	0.30 [0.08, 0.51]
SI → PU	0.62 [0.37, 0.86]	0.59 [0.37, 0.81]	0.59 [0.37, 0.82]	0.59 [0.37, 0.81]
BI → Freq	0.67 [0.60, 0.75]	0.67 [0.59, 0.74]	0.67 [0.60, 0.74]	0.67 [0.59, 0.74]
		Indirect		
PEOU→PU→BI	0.19 [–0.03, 0.40]	0.20 [0.04, 0.36]	0.20 [0.02, 0.38]	0.20 [0.04, 0.36]
SI→PU→BI	0.42 [0.28, 0.56]	0.39 [0.25, 0.54]	0.41 [0.27, 0.55]	0.40 [0.25, 0.54]
PU→BI→Freq	0.45 [0.28, 0.63]	0.45 [0.34, 0.55]	0.46 [0.35, 0.57]	0.45 [0.34, 0.55]
PEOU→PU→BI→Freq	0.13 [–0.02, 0.27]	0.13 [0.03, 0.24]	0.13 [0.01, 0.25]	0.13 [0.03, 0.24]
SI→PU→BI→Freq	0.28 [0.18, 0.38]	0.26 [0.16, 0.37]	0.27 [0.17, 0.37]	0.26 [0.16, 0.37]
TPC→BI→Freq	—	-0.14 [–0.21, –0.06]	—	−0.10 [−0.23, 0.02]
ST→BI→Freq	—	—	-0.11 [–0.18, –0.03]	−0.04 [−0.15, 0.07]

Note: Estimates are standardised with bootstrapped 95% CIs from 5,000 resamples.

Supplementary Section S5: Multiple Imputation Sensitivity Analysis Comparisons

Table S15. Multiple Imputation Sensitivity Analysis Comparisons

SI path	FIML β	MI β	MI range
Model 1: SI → BI	**0.21**	**0.17**	[0.14, 0.21]
Model 1: SI → PU	**0.62**	**0.60**	[0.52, 0.68]
Model 1: SI → PU → BI	**0.42**	**0.42**	[0.38, 0.47]
Model 1: SI→PU→BI→Freq	**0.28**	**0.28**	[0.25, 0.32]
Model 2: SI → BI	**0.07**	**0.07**	[0.02, 0.11]
Model 2: SI → PU	**0.59**	**0.58**	[0.50, 0.66]
Model 2: SI → PU → BI	**0.39**	**0.39**	[0.35, 0.46]
Model 2: SI→PU→BI→Freq	**0.26**	**0.26**	[0.23, 0.30]
Model 3: SI → BI	**0.11**	**0.10**	[0.05, 0.14]
Model 3: SI → PU	**0.59**	**0.58**	[0.50, 0.66]
Model 3: SI → PU → BI	**0.41**	**0.40**	[0.36, 0.46]
Model 3: SI→PU→BI→Freq	**0.27**	**0.27**	[0.24, 0.31]
Combined: SI → BI	**0.07**	**0.07**	[0.02, 0.11]
Combined: SI → PU	**0.59**	**0.58**	[0.50, 0.66]
Combined: SI → PU → BI	**0.40**	**0.39**	[0.35, 0.46]
Combined: SI→PU→BI→Freq	**0.26**	**0.26**	[0.23, 0.30]

Note. MI values are coefficients averaged across 20 imputed datasets, with min–max ranges.
